# Trends in Cognitive Function Among Chinese Elderly From 1998 to 2018: An Age-Period-Cohort Analysis

**DOI:** 10.3389/fpubh.2021.753671

**Published:** 2021-11-11

**Authors:** Xiaoqian Hu, Shuyan Gu, Xuemei Zhen, Xueshan Sun, Yuxuan Gu, Hengjin Dong

**Affiliations:** ^1^School of Politics and Public Administration, Qingdao University, Qingdao, China; ^2^Center for Health Policy Studies, School of Public Health, Zhejiang University School of Medicine, Hangzhou, China; ^3^Center for Health Policy and Management Studies, School of Government, Nanjing University, Nanjing, China; ^4^Center for Health Management and Policy, School of Public Health, Shandong University, Jinan, China; ^5^NHC Key Lab of Health Economics and Policy Research, Shandong University, Jinan, China; ^6^The Fourth Affiliated Hospital, Zhejiang University School of Medicine, Yiwu, China

**Keywords:** age-period-cohort, cognition, China, elderly, gender disparity

## Abstract

**Objectives:** To investigate the effects of age, period, and cohort (APC) on trends in cognitive function among the Chinese elderly, and to explore how gender gaps in cognitive function change with age, period, and cohort.

**Methods:** This study used data from the Chinese Longitudinal Healthy Longevity Survey (CLHLS) from 1998 to 2018, and included 90,432 participants aged above 65 years old. The measurement of cognitive function was the score of the Mini-Mental State Examination (MMSE). Cross-classified random-effect models were used to investigate age, period, and cohort trends in cognitive function.

**Results:** Mini-Mental State Examination scores decreased with age at an increasing rate. While the cohort effect was nearly stable, the period effect demonstrated a downward trend from 1998 to 2002 followed by a nearly flat line. Females were associated with lower MMSE scores than males. When age increased, the gender gaps in MMSE scores further increased. The period-based gender gaps in MMSE scores diverged throughout the 20 years, while the cohort-based gender disparities in MMSE scores converged with successive cohorts.

**Conclusions:** Age, period, and cohort had different and independent effects on cognitive function among the Chinese elderly. The effect of age was stronger than that of period and cohort. Gender disparities in cognitive function increased with age and period, and decreased with successive cohorts.

## Introduction

Population aging is one of the major challenges worldwide, such as in China (1). In 2020, there were 190.59 million people aged over 65 years old in China, accounting for 13.5% of the total population (2). In China, life expectancy at birth reached 76.62 years in 2015 (3). The elderly are relatively more vulnerable to cognitive impairment or dementia (4), which would place heavy care burdens on families and societies (5, 6). Therefore, the study on health among Chinese elderly, with expanded scales and increased longevity has important significance.

A better understanding of cognitive function trends is crucial to both the aging population and society because cognitive impairment will decrease the quality of life for the elderly and place care burdens on families of the elderly and social care systems (5, 7). Numerous studies have reported that cognitive function improved among the elderly in Sweden (8, 9), Denmark (10), Germany (11), the United States (12–14), and China (15). For example, US data suggested that the prevalence of cognitive impairment among the elderly decreased from 1993 to 2004 (14). However, other studies reported opposite trends for cognitive function. For example, findings from a Swedish study indicated that the cognitive function of individuals older than 77 years in 2002 was significantly worse compared with that of individuals interviewed in 1992 (16). Zeng determined that cognitive impairment increased among the Chinese elderly between 1998 and 2008 (1); whereas, these previous studies rarely adjusted for three unique effects related to health trends, which are age, period, and cohort (APC) effects. The effect of age reflects the biological and social processes of aging specific to individuals (17). The effect of period refers to external factors that simultaneously affect all age groups at a particular calendar time. The effect of period often results from shifts in social, technological, historical, and cultural environments, such as technology breakthroughs, world wars, famine, pandemics of infectious diseases, and public health interventions (18). For example, the advent and diffusion of new medical technology, which could prevent the spread of a kind of infectious disease, would reduce mortality rates of all age groups simultaneously (19). This example reflects period effects (20). The effect of cohort reflects different formative life experiences of successive generations (21, 22). Cohort effects subsume the effects of early life conditions and continuous exposure to socioeconomic, behavioral, and environmental factors that act persistently over time to produce differences in life course outcomes for specific cohorts (23). One example for the cohort effects would be, for instance, the research of Yang on happiness of Americans from 1972 to 2004 demonstrated that baby boomers experienced less happiness on average than both earlier and later cohorts. Yang explained that this may be closely related to early life conditions and formative experiences. The cohort of baby boomers experienced more competition to enter schools and the labor market because of larger cohort sizes, which might decrease the happiness level of the baby boomers (24). Furthermore, in the past several decades, China has experienced huge societal changes and rapid economic growth, and its people had undergone powerful social forces (15). These societal changes were expected to influence living conditions and the health of populations with distinct effects on different periods and birth cohorts. Since age, period, and cohort had distinct effects on health, these temporal sources of variations in cognitive function need to be distinguished (22).

To examine the cognitive function trends accurately, studies began to explore some of the APC effects which could be improved further. Wu et al. (25) reviewed 70 prevalent studies on dementia in mainland China, Hong Kong, and Taiwan from 1980 to 2012. They identified no significant variation across periods but a potentially increasing cohort effect. However, this study did not conduct complete APC modeling and robust statistical tests because of limited information and considerable variations across different studies. Another study in China reported that the prevalence of cognitive impairment remained stable from 1998 to 2014. Regarding the effect of cohort, cognitive impairment remained stable after a decline in early birth cohorts (25). While this study used the intrinsic estimator method for APC analyses, this method may be a poor approximation of the process of social change (18). Meanwhile, it only focused on the elderly aged 80 years and above, which may provide an incomplete picture of the effects of age. Given these limitations in the literature, studies using advanced methods to explore APC effects on cognitive function trends are required.

Substantial studies had demonstrated that women had worse cognitive function than men in cultural settings such as China (18). For both cultural and historical reasons, females were significantly disadvantaged in nutrition, education, and occupational achievement compared with their male counterparts in traditional China, all of which were associated with cognitive development and maintenance (26). While Chinese females experienced huge transitions and improvement in their living conditions and socioeconomic status across periods and cohorts in this century, the gender gaps in cognitive function may also change. However, few studies explored how gender differentials in cognitive function changed over APC among the Chinese elderly (22). Thus, it is necessary to explicate APC effects on trends of gender gaps in cognitive function, as these effects had been demonstrated in previous studies to be significant contributors to temporal health trends (19, 27).

Using data from the Chinese Longitudinal Healthy Longevity Survey (CLHLS), one of the best sources of data on Chinese elderly with multiple birth cohorts from 1998 to 2018, this study intends to investigate the effects of APC on trends of cognitive function among the Chinese elderly and delineate gender disparity trends of cognitive function by APC.

## Materials and Methods

### Data Sources

This study used data from the CLHLS, which focused on shedding new light on a better understanding of determinants of healthy longevity. Zeng and Vaupel (28) had introduced the survey design in detail previously. The baseline survey was conducted in 1998; seven follow-up surveys with replacements for deceased samples were conducted in 2000, 2002, 2005, 2008, 2011, 2014, and 2018. These surveys occurred in randomly selected counties and cities in 22 Chinese provinces (28). All centenarians (aged 100+ years) from these regions who voluntarily agreed to participate in the CLHLS were interviewed. For each centenarian, one nearby octogenarian (aged 80–89 years) and one nearby nonagenarian (aged 90–99 years) were matched and interviewed concerning gender and residence. From the 2002 wave, the CLHLS expanded the range of participants to those above 65 years old. Sample weights were made according to the age-gender-residence distribution of the elderly population (28). According to a previous assessment of the CLHLS, the quality of the CLHLS data was high regarding data completeness, reliability, and validity (29).

The CLHLS questionnaires comprised questions about basic information, self-reported life evaluation and personality, lifestyle, background, and cognitive function (28). The interview, along with some basic physical examinations, was conducted at the home or nursing institution of each participant by skilled interviewers. Every participant provided written informed consent before the survey. The Research Ethics Committees of Duke University and Peking University granted approval for the protection of human subjects for the CLHLS.

### Study Samples

Considering the CLHLS conducted over 20 years from 1998 to 2018 included the elderly across successive birth cohorts, it was suitable for the exploration of the APC effects on cognitive function trends. There were 92,860 participants aged over 65 years in all eight waves. As the self-reported age after 105 years old was not reliable (28), we excluded 2,202 participants aged over 105 years. Additionally, 226 participants were excluded, as they missed information on cognitive function. Thus, the final sample size in analyses was 90,432.

### Variables

#### Cognitive Function

To adapt to Chinese culture, cognitive function in this study was measured by the Chinese version of the Mini Mental State Examination (MMSE), which was modified based on the international standard MMSE questionnaire (30) and tested through pilot survey interviews (28). The MMSE is widely used in clinical and research settings to assess global cognitive function and screen for cognitive impairment (31). The Chinese version of the MMSE included items such as recall, orientation, language, reaction time, and calculation. The participants scored 1 for each correct answer (6). Thus, the range of MMSE score was from 0 to 30, with relatively high scores indicating better cognitive function.

#### Age, Period, and Cohort

For ease of interpretation of the intercept values, the age of the participants divided by 10 was the grand mean-centered (18). Period indicated the year the survey was conducted (1998, 2000, 2002, 2005, 2008, 2011, 2014, and 2018). Cohort was the year the participants were born. Participants who were born before 1900 or after 1940 were grouped separately to ensure a sufficient number of participants (32). We subsequent*ly grouped other birth cohorts into 5-year bands*.

##### Covariates

Gender (male = 0; female =1) is the key stratification factor to explore age-period-cohort effects on gender disparity in cognitive function. We also adjusted for the samples' demographic characteristics, socioeconomic status (SES), health behaviors, chronic diseases and prior test exposure (representing their associations with cognitive function in previous studies) (33). Demographic characteristics included ethnicity, current residence, birthplace, marital status, and co-residence. Ethnicity was defined as Han and minority (including all ethnic groups except Han). Current residence and birthplace were both dichotomized as urban and rural. Marital status was defined as married and not married (we combined divorced, separated, widowed, and never married as not married). Co-residence was defined as alone and living with others (such as household members and living in a nursing home). SES included education and job. Education was defined as illiterate (had not received any education) and literate. The CLHLS collected job information of the participants through the following question: “What was your main occupation before age 60?” Considering most people were farmers at that time, we defined job as farmers and others (such as professional and technical personnel; industrial worker; governmental, institutional, or managerial personnel; commercial or service worker; military personnel, and others). Health behaviors indicating the lifestyle of the participants at survey time included smoking, drinking, and physical exercise, which were dichotomized as yes and no. Chronic diseases included four common diseases among the elderly, hypertension, diabetes, heart disease, and stroke. Prior test exposure was used to adjust for potential practice effects of repeat cognitive function testing (14). Participants who had participated in previous waves of the survey was defined as “yes,” otherwise was defined as “no.”

### Statistical Methods

First, we summarized the basic characteristics of study samples in all the eight waves using means ± standard deviation or frequency (percentages).

We applied hierarchical APC (HAPC) models to simultaneously estimate the age, period, and cohort trends of the MMSE score for repeated cross-sectional data. The HAPC model was developed by Yang and her colleague, in which the three effects are not assumed to be additive at the same level of analysis (18). The HAPC model could address a classical APC identification problem in two ways (18). First, we grouped individuals born in a 5-year range into a single cohort to break the linear dependence among the dimensions of APC. Second, the nonlinear transformations approach suggested applying a parametric nonlinear transformation, such as polynomials, to at least one of the APC dimensions to break their linear relationships (24). According to this strategy and previous findings on curvilinear age effects on health (18), this study proposed models of MMSE scores as a quadratic function of age.

Thus, we fit HAPC cross-classified random effect regression models (HAPC-CCREMs) to examine the effects of APC on cognitive function. In each regression model, MMSE scores were regressed on age in linear and squared terms and other confounding variables as required. The coefficients of period, cohort, and gender were allowed to have random effects (24). This design made it possible to explore the period-based and cohort-based trends of gender gaps in cognitive function. In total, the model took the following form:

Level 1 model:


$$\begin{array}{l} MMSE_{ijk}=\;\beta_{0jk}+\beta_{1}A_{ijk}+\beta_{2}A_{ijk}^{2}+\beta_{3jk}S_{ijk}+\overset{P}{\underset{p=4}{\sum }}\beta_{p}X_{pijk}\\ \;+e_{ijk},\;e_{ijk}\;\sim \;N\left(0,\sigma^{2}\right) \end{array}$$

where *MMSE*_*ijk*_ stands for scores of MMSE for respondent *i* (for *i* = 1, 2, …, *n*_*jk*_) within period *j* (for *j* = 1, 2, …, 7) and cohort *k* (for *k* = 1, 2, …, 10); *A* and *A*^2^ denote age and age-squared, respectively; *S* denotes gender; *X*_*p*_ denotes the vector of other individual-level variables, such as age by gender, to test how the gender gap in cognitive function varies from age and covariates. β_0*jk*_ is the intercept indicating the cell mean for the reference group at the mean age interviewed in period *j* and belonging to cohort *k*; β_1_ and β_2_ denote the fixed coefficients for age; β_3*jk*_ denotes the random coefficients for gender; β_*p*_denotes the fixed coefficients for covariates; *P* is the maximum number of covariates included; *e*_*ijk*_ is the random individual effect or cell residual, which is assumed to be normally distributed with mean 0 and a within-cell variance σ^2^. Age divided by 10 is the grand mean-centered for ease of interpretation of the intercept values.

Level 2 model:


$$\begin{array}{l} \beta_{0jk}=\gamma_{0}+u_{0j}+v_{0k}\\ \beta_{3jk}=\gamma_{3}+u_{3j}+v_{3k} \end{array}$$

The level 2 models test whether with gender disparities in MMSE scores or not, varied by period or cohort through the specifications of random variance components for the random intercept and coefficients. β_0*jk*_ denotes a random intercept, which specifies that the overall mean varies from period to period and from cohort to cohort. γ_0_ is the expected mean at zero values of all level 1 variables averaged over all periods and cohorts; *u*_0*j*_ is the overall period effect regarding residual random coefficients of period *j* averaged over all cohorts with variance σ_*u*0_; *v*_0*k*_ is the overall cohort effect regarding residual random coefficients of cohort *k* averaged over all periods with variance σ_*v*0_. β_3*jk*_ denotes the random coefficients for gender; γ_3_ is the level 2 fixed-effect coefficient that represents the fixed effects of gender. To test whether the gender stratifications of MMSE scores varied by period or cohort, we specify that coefficients have period effects (*u*_3*j*_) and cohort effects (*v*_3*k*_) whose corresponding random variance components are σ_*u*3_ and σ_*v*3_. These random variance components of period and cohort for the intercept and coefficients are assumed to have multivariate normal distributions (24).

Therefore, in the level 1 model, we could test whether the gender disparity in MMSE scores varied with age by the interaction term of age with gender. The level 2 model could test whether this gap varied by period or cohort. Based on the combination of two-level models, we used six models to explore the effects of APC on trends of MMSE scores and change in gender disparities in MMSE scores with APC. Model 1 was a two-level model with a fixed effect for age and random effects for period and cohort to explore the net effects of APC on MMSE scores. Model 2 added the key independent variable, gender, to explore its influence on MMSE scores. Model 3 added the interaction between age and gender to explore how the gender disparity in MMSE scores varied with age. Model 4 adjusted confounding variables based on Model 3. Model 5 added random effects of coefficients of gender to explore how gender disparities in MMSE scores varied by period and cohort. Model 6 added covariates based on Model 5 to use a full model. Analyses were conducted using SAS PROC MIXED (18). Bayesian Information Criterion (BIC) was used to compare models concerning the goodness of fit, with a smaller BIC value indicating better model fit (34).

## Results

### Basic Characteristics of Samples

Table 1 presents basic characteristics of the samples in the eight surveys from 1998 to 2018. In total, there were 90,432 participants with an average age of 87.48 years old. Most of the respondents were Han, living with others, born in a rural area, illiterate, working as farmers, and not married. About 80% of the participants did not smoke and drink. The average MMSE score of all the samples was 21.8 and ranged from 20.79 to 23.12 among the eight surveys.

**Table 1:** Basic characteristics of samples in the eight surveys.

**Variables**	**ALL**	**1998**	**2000**	**2002**	**2005**	**2008**	**2011**	**2014**	**2018**
N	90,432	8,682	10,976	15,751	15,296	15,542	7,025	6,708	10,452
Age	87.48 ± 10.63	92.61 ± 7.46	91.36 ± 7.26	86.50 ± 11.44	86.26 ± 11.41	87.30 ± 11.02	85.31 ± 10.20	85.18 ±9.66	85.60 ±11.24
**Gender**									
Male	39,145 (43.3)	3,484 (40.1)	4,611 (42.0)	6,781 (43.1)	6,639 (43.4)	6,670 (42.9)	3,253 (46.3)	3,159 (47.1)	4,548 (43.5)
Female	51,287 (56.7)	5,198 (59.9)	6,365 (58.0)	8,970 (56.9)	8,657 (56.6)	8,872 (57.1)	3,772 (53.7)	3,549 (52.9)	5,904 (56.5)
**Residence**									
Urban	43,580 (48.2)	3,231 (37.2)	6,776 (61.7)	7,260 (46.1)	6,837 (44.7)	6,315 (40.6)	4,033 (57.4)	3,120 (46.5)	6,008 (57.5)
Rural	46,852 (51.8)	5,451 (62.8)	4,200 (38.3)	8,491 (53.9)	8,459 (55.3)	9,227 (59.4)	2,992 (42.6)	3,588 (53.5)	4,444 (42.5)
**Ethnicity**									
Han	84,972 (94.0)	8,056 (92.8)	10,311 (93.9)	14,895 (94.6)	14,368 (93.9)	14,578 (93.8)	6,636 (94.5)	6,216 (92.7)	9,912 (94.8)
Minority	5,460 (6.0)	626 (7.2)	665 (6.1)	856 (5.4)	928 (6.1)	964 (6.2)	389 (5.5)	492 (7.3)	540 (5.2)
**Marriage**									
Not married ^*^	63,458 (70.2)	7,251 (83.5)	8,892 (81.0)	11,021 (70.0)	10,520 (68.8)	10,881 (70.0)	4,410 (62.8)	4,069 (60.7)	6,414 (61.4)
Married	26,974 (29.8)	1,431 (16.5)	2,084 (19.0)	4,730 (30.0)	4,776 (31.2)	4,661 (30.0)	2,615 (37.2)	2,639 (39.3)	4,038 (38.6)
**Co-residence**									
With others	77,583 (85.8)	7,790 (89.7)	9,665 (88.1)	13,632 (86.5)	13,229 (86.5)	13,150 (84.6)	5,909 (84.1)	5,420 (80.8)	8,788 (84.1)
Alone	12,849 (14.2)	892 (10.3)	1,311 (11.9)	2,119 (13.5)	2,067 (13.5)	2,392 (15.4)	1,116 (15.9)	1,288 (19.2)	1,664 (15.9)
**Job**									
Famer	65,690 (72.6)	6,529 (75.2)	7,873 (71.7)	11,239 (71.4)	10,867 (71.0)	11,603 (74.7)	5,093 (72.5)	5,171 (77.1)	7,315 (70.0)
Others ^&^	24,742 (27.4)	2,153 (24.8)	3,103 (28.3)	4,512 (28.6)	4,429 (29.0)	3,939 (25.3)	1,932 (27.5)	1,537 (22.9)	3,137 (30.0)
**Education**									
Illiterate	54,483 (60.2)	5,868 (67.6)	6,997 (63.7)	9,679 (61.5)	9,270 (60.6)	9,732 (62.6)	3,915 (55.7)	3,720 (55.5)	5,302 (50.7)
Literate	35,949 (39.8)	2,814 (32.4)	3,979 (36.3)	6,072 (38.5)	6,026 (39.4)	5,810 (37.4)	3,110 (44.3)	2,988 (44.5)	5,150 (49.3)
**Birthplace**									
Urban	13,068 (14.5)	1,250 (14.4)	1,857 (16.9)	2,475 (15.8)	2,399 (15.7)	2,087 (13.4)	844 (12.0)	662 (9.9)	1,494 (14.3)
Rural	77,252 (85.4)	7,432 (85.6)	9,116 (83.1)	13,222 (84.2)	12,896 (84.3)	13,451 (86.6)	6,177 (88.0)	6,036 (90.1)	8,922 (85.7)
Missing^∧^	112 (0.1)	–	3 (<0.1)	54 (0.3)	1(<0.1)	4 (<0.1)	4 (<0.1)	10 (<0.1)	36 (0.3)
**Smoking**									
No	74,455(82.3)	7,196 (82.9)	9,131 (83.2)	12,832 (81.5)	12,318 (80.5)	12,856 (82.7)	5,717 (81.4)	5,577 (83.1)	8,828 (84.5)
Yes	15,966 (17.7)	1,484 (17.1)	1,845 (16.8)	2,919 (18.5)	2,978 (19.5)	2,686 (17.3)	1,308 (18.6)	1,131 (16.9)	1,615 (15.5)
Missing^∧^	11 (<0.1)	2 (<0.1)	–	–	–	–	–	–	9 (<0.1)
**Drinking**									
No	73,484 (81.3)	6,628 (76.4)	8,775 (79.9)	12,538 (79.6)	12,201 (79.8)	12,907 (83.0)	5,770 (82.1)	5,685 (84.7)	8,980 (86.0)
Yes	16,937 (18.7)	2,052 (23.6)	2,201 (20.1)	3,213 (20.4)	3,095 (20.2)	2,635 (17.0)	1,255 (17.9)	1,023 (15.3)	1,463 (14.0)
Missing^∧^	11 (<0.1)	2 (<0.1)	–	–	–	–	–	–	9 (<0.1)
**Physical exercise**									
No	62,416 (69.0)	6,349 (73.1)	7,320 (66.7)	10,731 (68.1)	10,563 (69.1)	11,280 (72.6)	4,252 (60.5)	4,851 (72.3)	7,070 (67.7)
Yes	28,005 (31.0)	2,331 (26.9)	3,656 (33.3)	5,020 (31.9)	4,733 (30.9)	4,262 (27.4)	2,773 (39.5)	1,857 (27.7)	3,373 (32.3)
Missing^∧^	11 (<0.1)	2 (<0.1)	–	–	–	–	–	–	9 (<0.1)
**Hypertension**									
No	69,319 (76.7)	7,313 (84.2)	9,221 (84.0)	13,087 (83.1)	12,193 (79.7)	12,365 (79.6)	4,799 (68.3)	4,345 (64.8)	5,996 (57.4)
Yes	21,112 (23.3)	1,369 (15.8)	1,755 (16.0)	2,664 (16.9)	3,103 (20.3)	3,177 (20.4)	2,226 (31.7)	2,362 (35.2)	4,456 (42.6)
Missing^∧^	1 (<0.1)	–	–	–	–	–	–	1 (<0.1)	–
**Diabetes**									
No	87,296 (96.5)	8,600 (99.1)	10,808 (98.5)	15,375 (97.6)	14,869 (97.2)	15,122 (97.3)	6,668 (94.9)	6,330 (94.4)	9,524 (91.1)
Yes	3,135 (3.5)	82 (0.9)	168 (1.5)	376 (2.4)	427 (2.8)	420 (2.7)	357 (5.1)	377 (5.6)	928 (8.9)
Missing^∧^	1 (<0.1)	–	–	–	–	–	–	1 (<0.1)	–
**Heart disease**									
No	80,214 (88.7)	7,916 (91.2)	10,021 (91.3)	14,235 (90.4)	13,726 (89.7)	14,055 (90.4)	5,965 (84.9)	5,749 (85.7)	8,547 (81.8)
Yes	10,217 (11.3)	766 (8.8)	955 (8.7)	1,516 (9.6)	1,570 (10.3)	1,487 (9.6)	1,060 (15.1)	958 (14.3)	1,905 (18.2)
Missing^∧^	1 (<0.1)	–	–	–	–	–	–	1 (<0.1)	–
**Stroke**									
No	84,354 (93.3)	8,345 (96.1)	10,485 (95.5)	14,848 (94.3)	14,369 (93.9)	14,572 (93.8)	6,391 (91.0)	6,071 (90.5)	9,273 (88.7)
Yes	6,077 (6.7)	337 (3.9)	491 (4.5)	903 (5.7)	927 (6.1)	970 (6.2)	634 (9.0)	636 (9.5)	1,179 (11.3)
Missing^∧^	1 (<0.1)	–	–	–	–	–	–	1 (<0.1)	–
**Prior test exposure**									
No	51,099 (56.5)	8,682 (100.0)	6,448 (58.7)	9,651 (61.3)	7,387 (48.3)	8,313 (53.5)	47 (0.7)	2,085 (31.1)	8,486 (81.2)
Yes	39,333 (43.5)	0 (0.0)	4,528 (41.3)	6,100 (38.7)	7,909 (51.7)	7,229 (46.5)	6,978 (99.3)	4,623 (68.9)	1,966 (18.8)
MMSE score	21.80 ± 9.23	21.15 ± 8.96	21.22 ± 9.11	22.02 ± 8.80	22.07 ± 9.31	20.79 ± 10.03	22.60 ± 8.84	23.12 ± 8.71	22.37 ± 9.22

*MMSE, Mini Mental State Examination. Data are presented as mean ± standard deviation or n (%)*.

∧ *Missing data were excluded from other percentage calculation*.

* *This category included divorced, separated, widowed, and never married*.

& *This category included professional and technical personnel; industrial worker; governmental, institutional or managerial personnel; commercial or service worker; military personnel, and others*.

### Age-Period-Cohort Trends and Differentials in MMSE Scores

**Figure 1:**
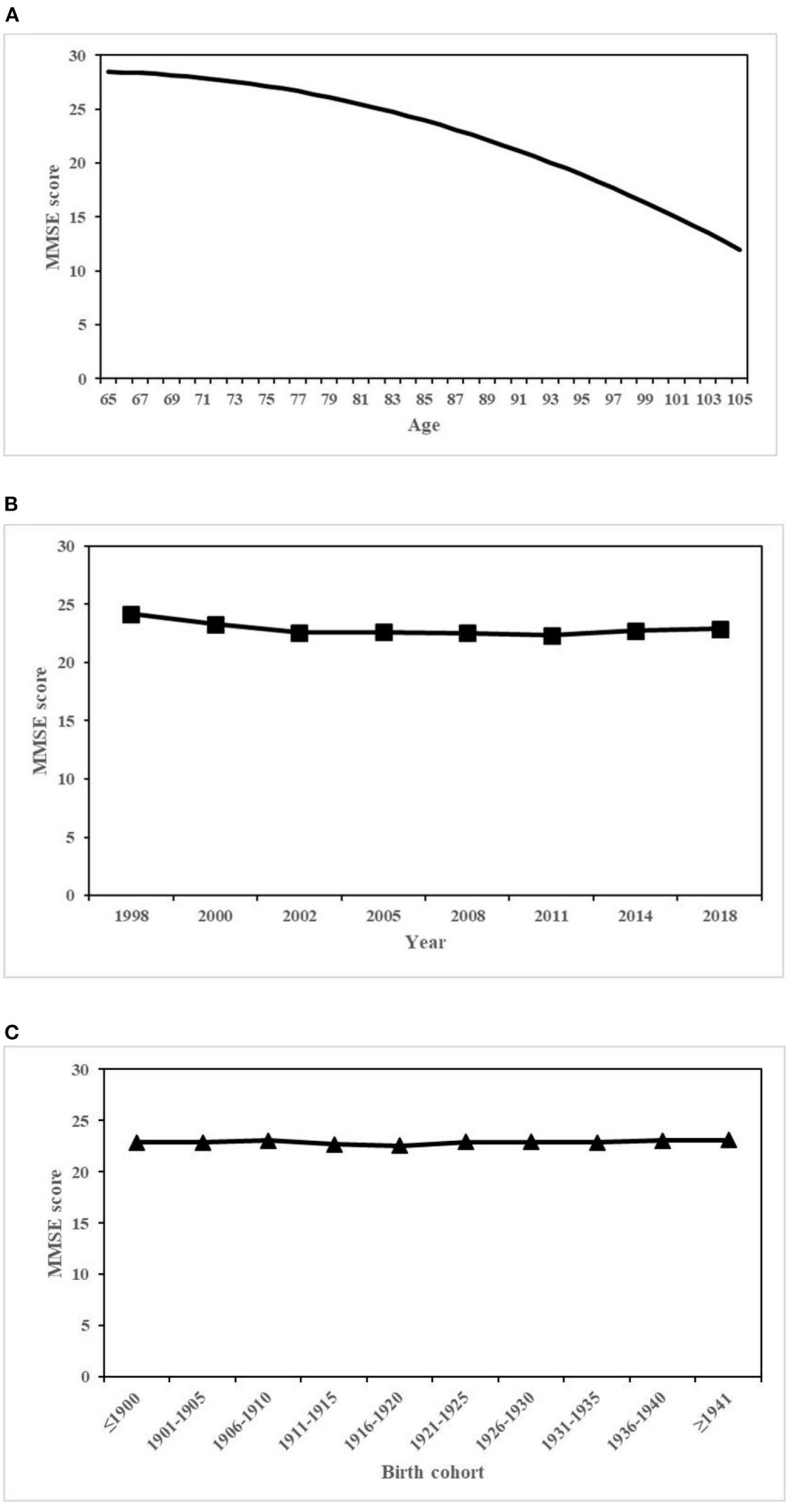
Overall age, period, and cohort effects on MMSE scores. **(A)** Age. **(B)** Period. **(C)** Cohort.

Table 2 presents estimates of fixed effects of all individual-level covariates and random-effect variance components. Model 1 showed that with only APC effects included in the model, the predicted average overall MMSE score was 22.9. Age had a significant negative quadratic effect on MMSE scores (coef. for age = −4.585, *p* < 0.001; coef. for age^2^ = −0.937, *p* < 0.001), which suggested that after period and cohort effects were taken into consideration, MMSE scores declined at an accelerated rate with age. Level 2 results suggested that MMSE scores varied in a smaller magnitude by period and cohort (coef. for period = 0.355, *p* = 0.034; coef. for cohort = 0.039, *p* = 0.081), relative to the effect of age. Figure 1 presents the overall trends of cognitive function in terms of predicted MMSE scores, estimated from model 1. Figure 1A showed curvilinear age effects. Figure 1B shows the effect of estimated period, which was calculated as ${\hat{\beta }}_{0j}={\hat{\gamma }}_{0}+u_{0j}$, where ${\hat{\gamma }}_{0}\;$was the intercept or estimated overall mean and *u*_0*j*_was the period-specific random-effect coefficients estimated from model 1. The effect of period demonstrated a downward trend from 1998 to 2002, followed by a nearly flat line. Figure 1C displays the estimated cohort effects in terms of the predicted MMSE scores at the mean age and averaged over all periods. Similar to the effect of period, the effect of cohort effect was calculated as ${\hat{\beta }}_{0k}={\hat{\gamma }}_{0}+v_{0k}$, where *v*_0*k*_ was the cohort-specific random-effect coefficients estimated from model 1. The effect of cohort was not significant, demonstrating a trend with little change.

**Table 2:** Hierarchical age–period–cohort cross-classified random-effect model estimates of MMSE scores.

	**Model 1**		**Model 2**		**Model 3**		**Model 4**		**Model 5**		**Model 6**	
	**Coef**.	**SE**	**Coef**.	**SE**	**Coef**.	**SE**	**Coef**.	**SE**	**Coef**.	**SE**	**Coef**.	**SE**
**Fixed effects**												
Intercept	22.899 ^***^	0.225	24.311 ^***^	0.262	24.368 ^***^	0.263	22.433^***^	0.264	24.337 ^***^	0.269	22.413^***^	0.251
Age	−4.585 ^***^	0.086	−4.597 ^***^	0.064	−4.018 ^***^	0.074	−3.462 ^***^	0.070	−4.165 ^***^	0.070	−3.449 ^***^	0.067
Age^2^	−0.937 ^***^	0.037	−1.015 ^***^	0.032	−0.944 ^***^	0.033	−0.942 ^***^	0.032	−0.958 ^***^	0.033	−0.931 ^***^	0.032
Gender (female = 1)			−2.159 ^***^	0.053	−2.241 ^***^	0.054	−0.868^***^	0.064	−2.107 ^**^	0.446	−0.872^***^	0.114
Age * Gender					−0.917 ^***^	0.052	−0.880 ^***^	0.051			−0.892 ^***^	0.055
Residence (rural = 1)							−0.174^**^	0.057			−0.170 ^**^	0.057
Ethnic (minority = 1)							0.969 ^***^	0.106			0.968 ^***^	0.106
Marriage (married = 1)							0.971 ^***^	0.070			0.974 ^***^	0.070
Living condition (alone = 1)							1.403 ^***^	0.076			1.403 ^***^	0.076
Job (farmer = 1)							−0.266 ^***^	0.070			−0.271 ^***^	0.070
Education (literate = 1)							1.611 ^***^	0.064			1.615 ^***^	0.064
Birthplace (rural = 1)							−0.589^***^	0.079			−0.584^***^	0.079
Smoking (yes = 1)							0.234^**^	0.073			0.233 ^**^	0.073
Drinking (yes = 1)							0.785^***^	0.069			0.785 ^***^	0.069
Physical exercise (yes = 1)							2.504^***^	0.058			2.508 ^***^	0.058
Hypertension (yes = 1)							0.257^***^	0.064			0.259 ^***^	0.064
Diabetes (yes = 1)							−0.261	0.142			−0.260	0.142
Heart disease (yes = 1)							0.018	0.083			0.020	0.083
Stroke (yes = 1)							−2.976^***^	0.103			−2.980 ^***^	0.103
Prior test exposure (yes = 1)							−0.185^**^	0.060			−0.187 ^**^	0.060
**Variance components**												
**Period**												
Intercept	0.355 ^*^	0.194	0.453 ^*^	0.248	0.451 ^*^	0.248	0.380^*^	0.210	0.482 ^*^	0.272	0.340 ^*^	0.191
Gender									0.314 ^*^	0.185	0.062	0.043
**Cohort**												
Intercept	0.039	0.028	0.087 ^*^	0.051	0.098 ^*^	0.056	0.074 ^*^	0.043	0.089	0.072	0.062	0.038
Gender									1.563 ^*^	0.757	0.007	0.015
**Model fit**												
BIC	708,261.5		628,065.5		627,755.3		622,020.3		627,809.3		621,999.8	

*MMSE, Mini Mental State Examination; SE, standard error; Coef., coefficient; BIC, Bayesian Information Criterion*.

* *p ≤ 0.05*;

** *p ≤ 0.01*;

*** *p ≤ 0.001*.

### Age-Period-Cohort Trends of Gender Disparities in MMSE Scores

**Figure 2:**
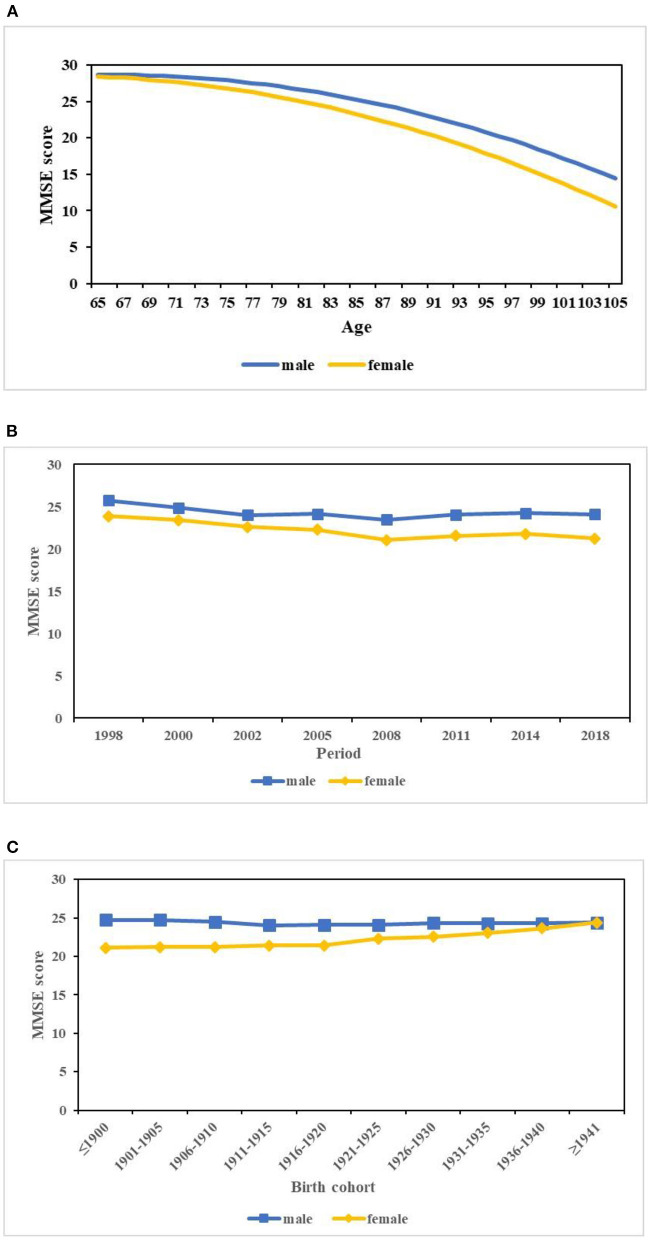
Predicted age, period, and cohort trends in the gender disparity in MMSE scores. **(A)** Age. **(B)** Period. **(C)** Cohort.

Model 2 indicated that the females had significantly lower MMSE scores (coef. = −2.159, *p* < 0.001) relative to the males when the effects of APC were considered. Model 3 indicated that the gender disparity in MMSE scores varied significantly with age (coef. = −0.917, *p* < 0.001). When age increased, the gender gap further increased (Figure 2A). Model 4 revealed that rural residence, ethnicity, marital status, living condition, job, education, birthplace, smoking, drinking, physical exercise, hypertension, stroke, and prior test exposure had significant influences on MMSE scores. Those participants who were born in a rural area, illiterate, farmers, not in a marriage, living with others, and suffering from stroke had lower MMSE scores. Comparing model 3 with model 4, the interaction effect of gender with age remained highly significant but decreased a little in size when confounding variables were considered.

When it came to the effects of period on gender disparities in MMSE scores, model 5 demonstrated that the gender gap in MMSE scores varied significantly with period net of age and cohort (coef. = 0.314, *p* = 0.046). Figure 2B displays the estimated random period effects on gender disparities in MMSE scores, which was calculated as ${\hat{\beta }}_{0j}+{\hat{\beta }}_{3j}={\hat{\gamma }}_{0}+{\hat{\gamma }}_{3}+u_{0j}+u_{3j}$ (where ${\hat{\gamma }}_{0}\;$was the intercept or estimated overall mean, ${\hat{\gamma }}_{3}\;$was the estimated fixed gender effect coefficient, *u*_0*j*_ was the period-specific random-effect coefficients, and *u*_3*j*_ was the gender-specific random period effects). Figure 2B shows that the gap in MMSE scores between the males and females further increased throughout the 20 years. Although the MMSE scores for both genders indicated decreasing trends, those of the females declined more.

Cohort effects on the gender disparity in MMSE scores were also significant when age and period were considered (coef. = 1.563, *p* = 0.019) (model 5 in Table 2). Figure 2C displays the estimated random cohort effect on gender disparities in MMSE scores, which was calculated as ${\hat{\beta }}_{0k}+{\hat{\beta }}_{3k}={\hat{\gamma }}_{0}+{\hat{\gamma }}_{3}+v_{0k}+v_{3k}$ Similar to the period effect, *v*_0*k*_ was the cohort-specific random-effect coefficient and *v*_3*k*_ was the gender-specific random cohort effect. From Figure 2C, we identified that the gap in MMSE scores between genders decreased across cohorts, which was largely because of the increasing trend of MMSE scores for females and the relatively stable trend for males among successive cohorts. Model 6, the final model, showed that period and cohort effects on gender differentials in MMSE scores were not statistically significant when covariates were taken into account.

## Discussion

Using eight waves of the CLHLS data from 1998 to 2018, we applied HAPC-CCREMs to explore trends of cognitive function among the Chinese elderly. Our findings indicated that the MMSE scores decreased with age at an accelerated rate. While the effect of cohort was nearly stable, the effect of period demonstrated a downward trend from 1998 to 2002 followed by a nearly flat line. The females were associated with lower MMSE scores than males. When age increased, the gender gap in cognitive function further increased. The period trends of gender gaps in MMSE scores widened throughout the 20 years, while the cohort trends of gender disparities in MMSE scores narrowed with successive cohorts.

The results of the APC model analysis indicated that the effect of APC on cognitive function among the Chinese elderly were distinct and independent of each other. These different effects suggested that it is of vital importance to test variations formally in all three time-related dimensions in studies on trends in health (24). In line with previous studies (22), we identified that the MMSE scores decreased with age at an accelerated rate. Compared with the effect age, the effect of period and cohort was smaller. The effect of period demonstrated a downward trend from 1998 to 2002, followed by a nearly flat line. The downward trend may partly be because of in 1998 and 2000 waves of the survey, CLHLS mainly focused on elders above 80 years old. Those oldest-olds who could survive to advanced ages usually had relatively better health status (35), resulting in higher MMSE scores in the 1998 and 2000 waves than scores in the follow-up waves of survey including elders above 65 years old. The stable period trend from 2002 to 2018 was consistent with previous research (22). Zhang also determined that with the net of age and cohort effect, the period-based trend among the Chinese elderly was relatively stable (22). The effect of cohort was not significant, demonstrating a trend with little change from our results, which were different from another Chinese study (22). According to the results of Zhang, cognitive impairment declined across birth cohorts. Zhang used the rate of cognitive impairment as the dependent variable, while our studies used scores of cognitive function, including both the rate and severity of cognitive impairment, which may have different results.

We identified that the females had significantly worse cognitive function than the males, consistent with the results from Taiwan (36) and India (37) but different from the results from developed countries (31, 38). This may be due to the long-lasting preference for sons in traditional Chinese society; compared with males, most females had a relatively tough early life with bad nutrition (33) and few opportunities to obtain education (39), all of which were associated with disadvantages in cognitive development and maintenance (26). Furthermore, this gender disparity in cognitive function further increased with increase in age from our results, supporting the cumulative disadvantage theory (40). According to the cumulative disadvantage theory, early disadvantages would accumulate over the life course by setting people onto different life trajectories, resulting in increasing disparities in health as people age (33). Considering the bad cognitive function and longevity of females, more attention and resources should be given to them to ensure they obtain adequate care.

Gender disparities in MMSE scores enlarged significantly from 1998 to 2018. Although the MMSE scores for both genders indicated decreasing trends, those of the females declined more. Similarly, Zeng determined that the elderly had lower MMSE scores than those of the same age interviewed 10 years ago; meanwhile, the cognitive function of females declined faster than that of males (1). This finding could be explained by the expansion of the morbidity theory, indicating that improvements in medical conditions and living standards in recent years may result in some frail elderly individuals being saved from dying but surviving with poor cognitive function, which may reduce the whole scores of cognitive function (10).

We observed that the cohort trends of gender disparities in cognitive function narrowed with cohorts, which may largely be because of the increase in MMSE scores for females among successive cohorts. The improvement in cognitive function for females in late-born cohorts that we found was consistent with previous studies (22, 41). We speculated that the decreasing gender gap in cognitive function with cohort could be substantially explained by differential exposures to various social correlates of cognitive function, especially the access of women to schooling had improved significantly over time in China (42). Numerous studies from different countries had demonstrated the association between education and late-life cognition as measured by cognitive tests, cognitive impairment, or dementia (33). Education would not only promote cognitive development in early life, but also lead to higher SES, better living condition, and more cognitive reserve in later life (26, 43). Thus, public policy targeting education is required, which will not only improve the lives of children but also enhance cognitive well-being and bridge the gender gaps of the elderly ultimately.

While our results provided new insights into the trends of cognitive function among the Chinese elderly, there were some limitations. First, we performed MMSE to evaluate cognitive function rather than comprehensive clinical evaluations. MMSE is a screening tool and provides global cognitive function. The clinical evaluations are more accurate; hence further analyses by different domains of cognitive function are needed. Second, because of data limitation, the earliest and latest birth cohorts did not capture a full age distribution, which may bias the estimates for cohort trends. Third, we focused on the basic effects of APC and individual-level variables in this study; effects from macroeconomic and medical variables on cognitive function should be further explored.

In conclusion, we assessed the trends of cognitive function among the Chinese elderly using the APC model. The gender gaps in cognitive function increased with age and period but decreased with cohorts. The significance of APC effects in shaping social inequalities in cognitive function implied the relevance of both biological forces and historical context. These findings might help inform healthcare planning and priorities for medical resource allocation accordingly.

## Data Availability Statement

Publicly available datasets were analyzed in this study. This data can be found here: https://sites.duke.edu/centerforaging/programs/chinese-longitudinal-healthy-longevity-survey-clhls/.

## Ethics Statement

The studies involving human participants were reviewed and approved by the Research Ethics Committees of Duke University and Peking University. The patients/participants provided their written informed consent to participate in this study.

## Author Contributions

XH, SG, XZ, XS, YG, and HD contributed to study conception and design. Material preparation and analysis were performed by XH, SG, XZ, XS, and YG. The first draft of the manuscript was written by XH. All authors commented on previous versions of the article. All authors contributed to the article and approved the submitted version.

## Funding

This study was funded by the National Natural Science Foundation of China (Grant No.: 71490732).

## Conflict of Interest

The authors declare that the research was conducted in the absence of any commercial or financial relationships that could be construed as a potential conflict of interest.

## Publisher's Note

All claims expressed in this article are solely those of the authors and do not necessarily represent those of their affiliated organizations, or those of the publisher, the editors and the reviewers. Any product that may be evaluated in this article, or claim that may be made by its manufacturer, is not guaranteed or endorsed by the publisher.
